# In Silico Genome-Wide Profiling of Conserved miRNAs in AAA, AAB, and ABB Groups of *Musa* spp.: Unveiling MicroRNA-Mediated Drought Response

**DOI:** 10.3390/ijms26136385

**Published:** 2025-07-02

**Authors:** Kishan Saha, Onyinye C. Ihearahu, Vanessa E. J. Agbor, Teon Evans, Labode Hospice Stevenson Naitchede, Supriyo Ray, George Ude

**Affiliations:** Department of Natural Sciences, Bowie State University, 14000 Jericho Park Road, Bowie, MD 20715, USA; ksaha@bowiestate.edu (K.S.); oihearahu@bowiestate.edu (O.C.I.); vagbor14@gmail.com (V.E.J.A.); evanst0415@students.bowiestate.edu (T.E.); lnaitchede@bowiestate.edu (L.H.S.N.); sray@bowiestate.edu (S.R.)

**Keywords:** *Musa* spp., in silico, miRNAs, target genes, qRT-PCR, drought stress

## Abstract

Small non-coding microRNAs (miRNAs) play crucial roles in the degradation of the messenger RNAs (mRNAs) that are involved in various biological processes post-transcriptionally and translationally. Many plants, especially *Musa* spp. (plantains and bananas), which are important perennial herbs of the family Musaceae, experience significant yield loss due to abiotic stressors, yet only a few miRNAs involved in this response have been identified. This study employed in silico analyses of transcriptome shotgun assembly (TSA) and expressed sequence tag (EST) sequences to identify *Musa* miRNAs and their target genes. Leaf and root tissues from three *Musa* genomic groups (AAA, AAB, and ABB) under drought stress were analyzed using quantitative real-time PCR (qRT-PCR) to validate the expression of miRNAs. A total of 17 potential conserved miRNAs from 11 families were identified, with the minimal folding free energies (-kcal/mol) of precursors ranging from −136.00 to −55.70, as observed through RNA folding analysis. Six miRNAs (miR530-5p, miR528-5p, miR482a, miR397a, miR160h, and miR399a) showed distinct tissue-specific expression patterns in the roots and leaves across the three groups. A total of 59 target regulatory transcription factors and enzymes involved in stress response, growth, and metabolism were predicted. Of these, 11 targets were validated for miR530-5p, miR528-5p, miR482a, and miR397a, using qRT-PCR. These four stress-responsive miRNAs exhibited an inverse expression relationship with their target genes across two different tissues in *Musa* groups. This research provides insights into miRNA-mediated drought stress responsiveness in *Musa* spp., potentially benefiting future studies on gene regulation under drought stress.

## 1. Introduction

MicroRNAs (miRNAs) are non-coding, single-stranded small RNAs with a length of 20–24 nucleotides (nt). They are crucial regulators of gene expression guided by the RNA-induced silencing complex (RISC) to target mRNAs for cleavage and transcriptional repression [1,2,3]. They also act as the regulators of a variety of cellular processes, such as plant development, stress adaptation, and various physiological responses by modulating transcription factors (TFs) and stress-responsive proteins in extreme environmental conditions [4,5,6]. The process of miRNAs starts from MIR genes that are transcribed to form long primary transcripts (pri-miRNAs) whose structures are sequentially cleaved by the DICER-LIKE 1 (DCL-1) protein into miRNA/miRNA* duplexes (pre-miRNA) [7,8]. Further, pre-miRNA is exported to the cytoplasm, where one strand is loaded into AGO1 to form RISC, which guides target mRNA cleavage or repression [9]. However, advances in high-throughput sequencing and bioinformatics have enabled the comprehensive cataloging of both conserved and species-specific miRNAs, offering insights into their evolutionary dynamics and functions [10]. Researchers can predict miRNA precursor hairpins, assess secondary structures, and map target interactions by integrating sequencing data from public databases such as transcriptome shotgun assembly (TSA), expressed sequence tags (ESTs), and genomic survey sequences (GSSs) with the miRNA database and repositories [11,12,13,14,15]. These approaches revealed conserved miRNA families involved in regulating critical key developmental and stress response pathways, as well as species-specific miRNAs linked to unique adaptive traits [16,17]. In recent years, in silico-based approaches have been extensively employed to analyze regulatory networks mediated by microRNAs (miRNAs) in several plant species including *Macrotyloma uniflorum* [14], *Oryza sativa* [18,19], *Nicotiana* spp. [20], *Chenopodium quinoa* [21], *Corchorus capsularis* [22], *Saccharum officinarum* [23], and *Vigna unguiculata* [24]. Like other crops, bananas and plantains (*Musa* spp.) play a vital role in the diets of millions of people in tropical and subtropical regions due to their high nutritional value, providing essential vitamins, minerals, and dietary fiber [25,26]. However, several abiotic factors, including drought, disrupt photosynthesis and hinder fruit development, leading to a significant yield loss of *Musa* plants [27,28]. Interestingly, *Musa* spp. with a B genome are known to be more resistant to abiotic stress than those with only an A genome. Notably, *Musa* spp. with an ABB genome demonstrated enhanced tolerance to drought and other environmental challenges [29]. The spatial expression of miRNAs and their target genes revealed that root-specific miRNAs fine-tune local adaptation by targeting the transcription factors (TFs) involved in osmotic balance and root architecture, while leaf-enriched miRNAs modulate stomatal regulation and photosynthetic gene networks [30,31]. Despite these insights, in silico analyses using expressed sequence tags (ESTs) and genome survey sequences (GSSs) in various *Musa* accessions have thus far yielded only a limited repertoire of conserved miRNAs and their predicted targets [11,13]. Previously, high-throughput small RNA sequencing in *Musa itinerans* identified over 250 conserved miRNAs and a few novel miRNAs under cold stress [32]. The homologs of drought-responsive miRNAs such as miR159, miR164, miR169, and miR398 exhibited significant up- or downregulation during water-deficit treatments in several plant species [33]. It was noted that the overexpression of native *Musa*-miR397 enhanced biomass but did not compromise tolerance towards copper (Cu) deficiency and sodium chloride (NaCl) stress [34]. In a study involving the *Musa acuminata* (calcutta-4) variety, treatment with *Pseudocercospora musae* led to the identification of 202 conserved miRNAs and 24 predicted novel miRNAs using Illumina sequencing [35]. Nevertheless, comprehensive genome-wide studies focusing on drought-responsive miRNA discovery, functional characterization, and the validation of their target genes across diverse *Musa* genotypes remain inattentive. In silico approaches have thus proven exceptionally versatile by integrating computational tools with high-throughput data, which can be utilized to analyze miRNAs across diverse species.

This study aimed to identify and characterize miRNAs in three genomic (AAA, AAB, and ABB) groups of *Musa* spp. using the TSA and EST datasets. Additionally, drought stress treatment was applied to explore the expression patterns of stress-responsive miRNAs and their target genes. The expression data provided valuable insights into miRNA-mediated drought responses, contributing to a deeper understanding of essential biological processes. These findings support the development of genetic engineering strategies that aim to enhance stress tolerance and disease resistance, particularly in *Musa* spp., thereby promoting sustainable agricultural practices.

## 2. Results

### 2.1. Identification of Potential miRNAs in Musa *spp.*

The identification and characterization of the conserved miRNAs of *Musa* spp. were employed using TSA- and EST-based sequences. Different in silico approaches were used to finalize the putative miRNAs with specific gene targets in *Musa* spp. Significant miRNA homologs of *Musa* were identified using 5510 previously known non-redundant miRNAs of Viridiplantae by executing BLASTn analysis with 38,128 TSA contigs and 45,771 EST sequences of *Musa*. After applying stringent filtering (<2 mismatch and length > 17 nt) and removing duplicate sequences of IDs, 146 TSA and 85 EST sequences were identified (Table S1) as putative candidate sequences with non-coding regions. Following the removal of protein-coding sequences from the TSA and EST datasets, a total of 231 (146 TSAs and 85 ESTs) non-coding sequences were used to predict precursor microRNAs corresponding to 231 conserved miRNAs in *Musa* spp. Further, validation was conducted by generating the secondary structure of the predicted precursor miRNA (pre-miRNA) of *Musa* spp.

### 2.2. Prediction and Validation of Secondary Structure of Pre-miRNA

**Figure 1:**
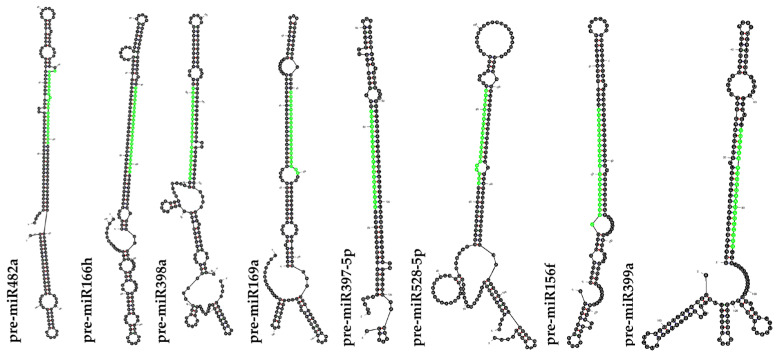
RNA secondary structure of predicted precursor miRNAs of *Musa* spp. determined by RNA folding analysis. Lime green color represents the predicted Musa miRNA sequences.

**Figure 2:**
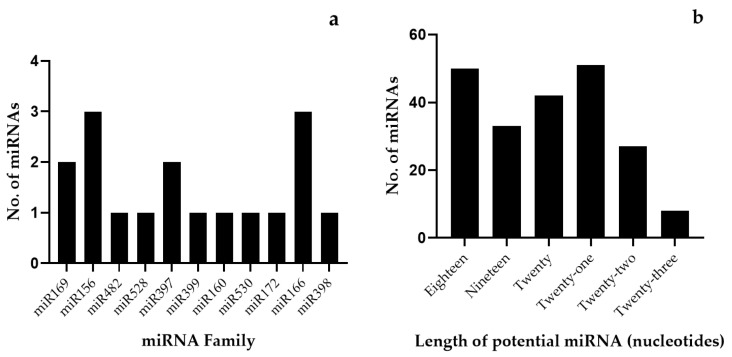
Distribution and length variations in identified *Musa* microRNA families. (**a**) Highlighting number of miRNA members and overall diversity across detected families and (**b**) length variations in mature miRNAs and frequency of nucleotide lengths observed from TSA and EST sequences.

To distinguish the miRNAs from other small RNAs such as tRNAs, rRNAs, and mRNAs, various structural analyses were performed. The Mfold analyses depicted that the most advanced feature of the stable secondary RNA structure with its minimal folding free energy (MEF) ranging from −51.3 to −88.40 (kcal mol^−1^) was observed in 17 predicted precursor sequences (Table 1). The minimum free energy index (MEFI) was found to be 0.69–2.25. The percentage of the AU and GC content of pre-miRNA ranged from 38.12 to 68.33 and from 31.67 to 61.88, respectively. The average length of pre-miRNA was found to be 107 nt in all 17 miRNAs studied. After carefully analyzing the stem-loop structure (Figure 1), 17 conserved putative *Musa* miRNAs were confirmed. These 17 miRNAs belonged to 11 distinct families, including miR169, miR156, miR482, miR528, miRNA397, miR399, miRNA160, miRNA530, miRNA172, miRNA166, and miR398 (Table 2). Of these, eleven miRNAs were predicted from ESTs and six miRNAs from TSA sequences (Figure 2).

### 2.3. Conservation and Phylogenetic Analysis

**Figure 3:**
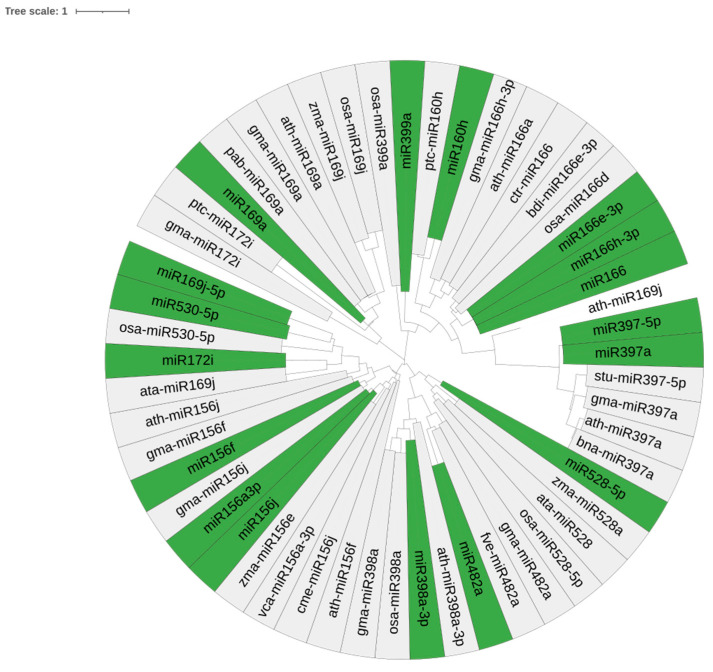
Phylogenetic tree analysis of predicted precursor *Musa* miRNAs with their closely related plant-specific precursor miRNAs generated using MEGA7 and annotated in iTOL web-based program. miRNAs observed in *Musa* spp. are highlighted in green.

The identified pre-miRNA homologs were retrieved by performing a BLAST against the miR database. The filtered sequences of the same family were employed for phylogenetic tree analysis. A high conservation of the nucleotide sequences of the pre-miRNA of *Musa* was observed (green) with other conserved plant precursor miRNA sequences. Thus, the phylogenetic tree showed that *Musa* miRNAs shared similarities with monocots like *Zea mays*, *Glycine max*, and *Oryza sativa* (Figure 3). Notably, *Musa* miRNAs appear to have evolved at different rates in various periods, similarly to other plant species.

### 2.4. Prediction of miRNA Target Genes and Functional Analysis

**Figure 4:**
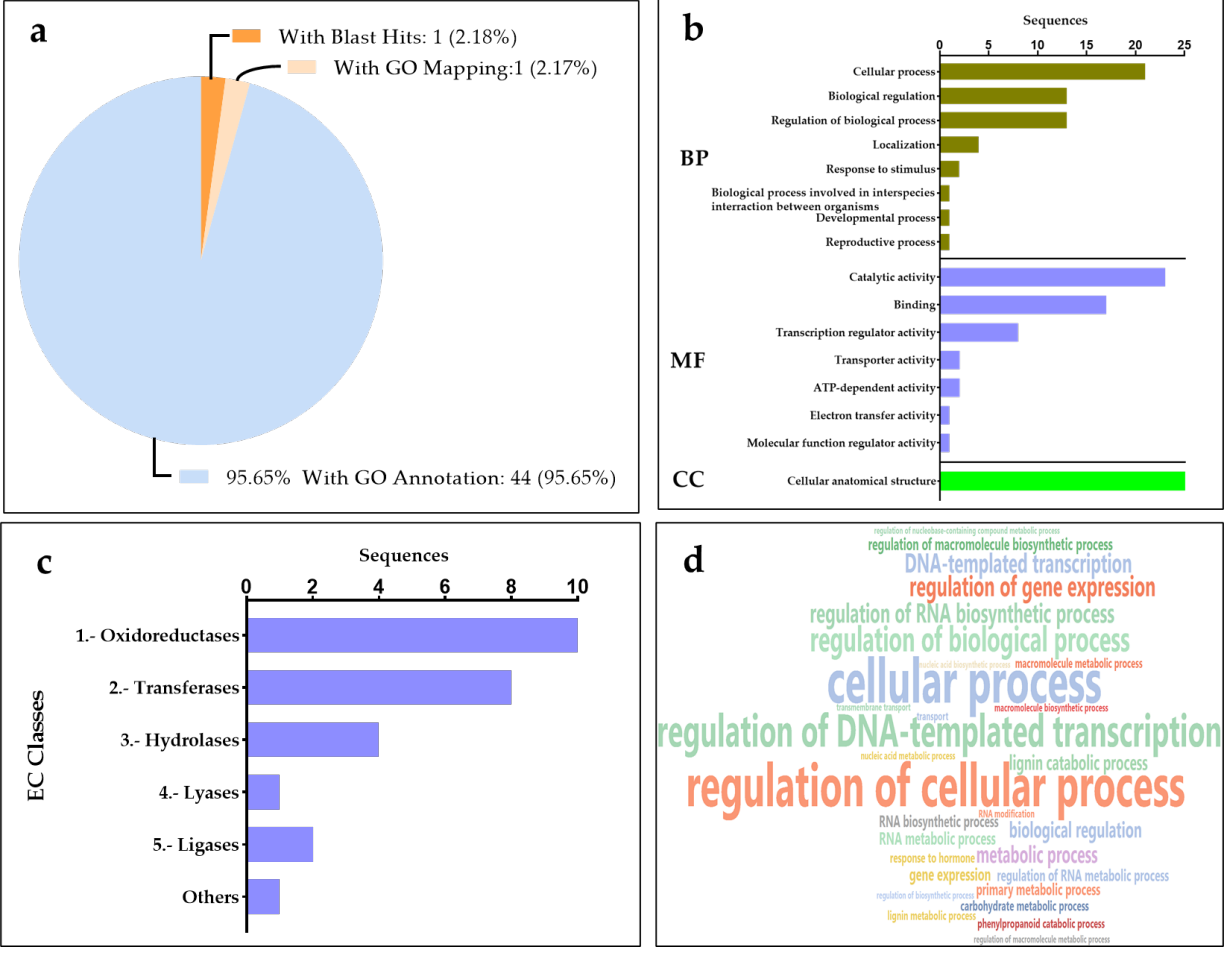
Target groups categorized by functional category identification using OmicsBox. (**a**) Pie chart showing sequence distribution of data with percentages of GO annotation, blast hits, and GO mapping. (**b**) Gene ontology analysis of target genes (number of sequences) in terms of biological process (BP), molecular function (MF), and cellular components (CC). (**c**) Distribution of enzyme codes and the corresponding number of sequences identified through functional analysis. (**d**) Functional enrichment of cellular components of *Musa* miRNA target genes.

MicroRNAs (miRNAs) play critical roles in the regulation of gene expression by binding to complementary mRNA sequences in their target genes. In this study, we obtained these target gene sequences from the Banana Genome Hub and identified potential miRNA target sites using the well-established psRNATarget tool. Using an E-value cutoff of ≤3 for miRNA target gene prediction, 59 target genes for 17 miRNAs were identified. The targets were classified into different functional categories and found to be inhibited through either cleavage or translational repression (Table S2). It was observed that highly conserved miRNA family target genes included LRR receptor-like serine/threonine protein kinases, poly-galactourinases, ABC transporter, NBS-LRR class resistant protein, RGA2 protein, polyphenol oxidase, NAC transcription factors, mavicyanin, and laccase. Subsequently, the selected miRNA target genes were mostly associated with metabolism, stress responses, transcription factors, growth development, and signal transduction in several plant species, including *Musa* spp. (Table 3). Furthermore, a GO enrichment analysis with 59 target genes was performed, which were mainly enriched in terms involved in biological processes such as the regulation of DNA template transcription factors, the carbohydrate metabolic process, cell signaling, and transmembrane transport in *Musa* spp. (Figure 4). For molecular function, the genes are mostly enriched in DNA binding, oxidoreductase activity, transferase activity, transcription regulator activity, and hydrolase activity. Within the cellular component category, the target genes were commonly enriched in terms of the nucleus, extracellular region, endoplasmic reticulum, membrane, and intracellular organelles in *Musa* spp. (Figure 4).

**Figure 5:**
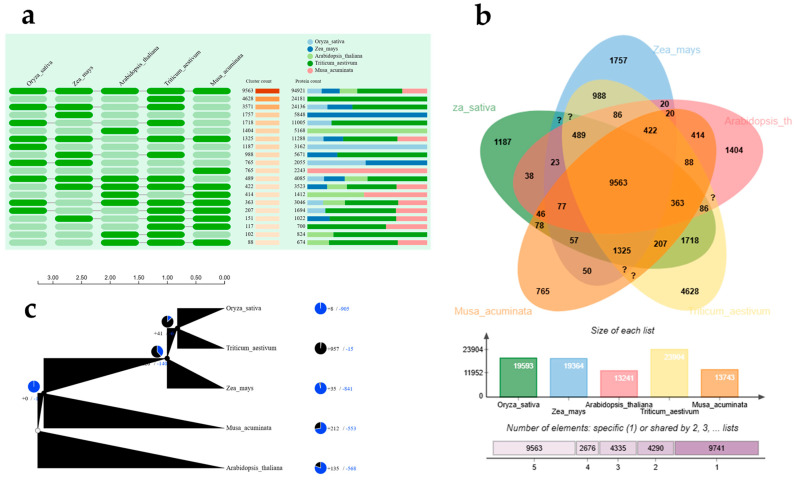
Predicted miRNA target homologs across species that were identified using OrthoVenn2. (**a**) Pattern of shared orthologous groups amongst *Oryza sativa*, *Zea mays*, *Arabidopsis thaliana*, *Triticum aestivum*, and *Musa acuminata*. Cluster count and protein count indicate number of clusters and proteins, respectively, shared between species. (**b**) Distribution of shared orthologous clusters among species of *Oryza sativa* (za_Sativa), *Zea mays*, *Arabidopsis thaliana* (Arabidopsis_th), *Triticum aestivum*, and *Musa acuminata*. (**c**) Pie charts show number of gene families that have expanded (black) or contracted (blue) during evolution, while phylogenetic trees depict evolutionary timelines of species.

In addition, a genome-wide comparison analysis with 59 target proteins was performed using the Orthovenn platform to identify and visualize orthologous clusters across multiple species. The data analysis resulted in a total of 9563 clusters of proteins being identified. The number of clusters for each taxon was varied, i.e., 19,593 for *Oryza sativa*, 19,364 for *Zea mays*, 13,241 for *Arabidopsis thaliana*, 23,904 for *Triticum aestivum*, and 13,743 for *Musa acuminata*. The phylogenetic tree depicted 212 gene families that were newly included upon the evolution of the species itself (Figure 5).

### 2.5. Expression Analysis of miRNAs and Their Target Genes in Drought-Stressed Plants

**Figure 6:**
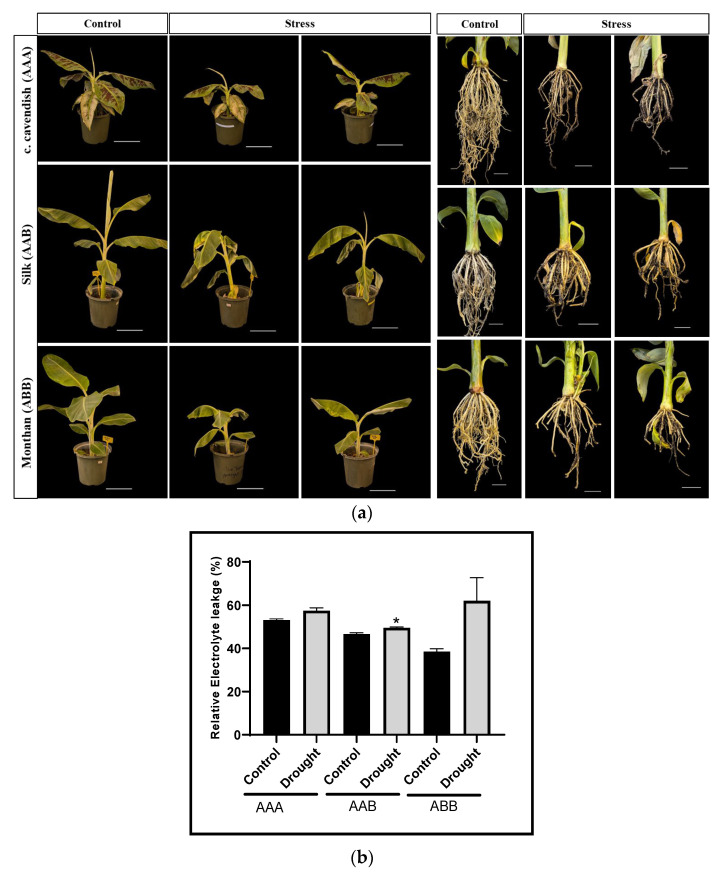
Phenotypic comparison of three groups of *Musa* spp. under control and drought conditions. (**a**) Left three panels display whole plants after 20 days of drought stress, along with regularly watered control plants showing wilting of leaves. Right three panels illustrate root morphology, indicating fewer root mats in drought stress compared to control. Scale bar = 5 cm. (**b**) Electrolytic leakage (% of total electrolyte leakage) measured in control and drought-stressed samples. Data represent membrane damage under drought stress, with higher values indicating increased cellular damage. Error bar showing six replicates of standard error of means. Asterisks indicate significant differences at *p* < 0.05.

Total RNA was extracted from the leaves and roots of drought-stressed and control plants. To confirm the effectiveness of the drought treatment, soil moisture content was monitored, verifying that the stressed plants experienced a deficit in water content (Figure S1). Additionally, electrolyte leakage assay quantitatively revealed increased ion leakage in the drought-stressed plants, indicating impaired membrane integrity (Figure 6). Phenotypically, the effects of drought stress were evident in the plants, which showed noticeable differences in root and leaf morphology; stressed plants exhibited wilting compared to the control group (Figure 6). Additionally, a reduction in root mats was observed in the drought-stressed plants relative to the controls. Further, cDNA was synthesized for target gene analysis using oligo (dT) and stem-loop primers that were designed to conduct the expression analysis of miRNAs through qRT-PCR.

**Figure 7:**
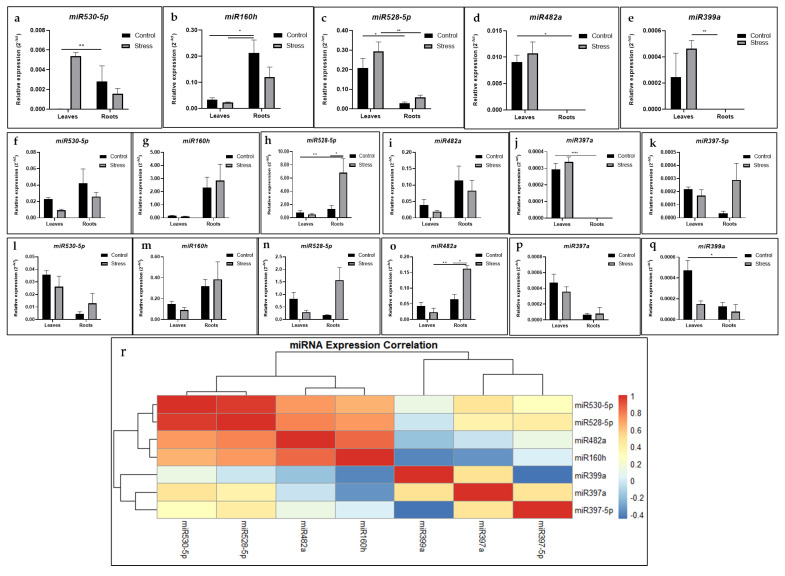
Effects of drought stress on expression of several conserved *Musa* miRNAs in leaves and roots of three different genomic groups AAA (**a**–**e**), AAB (**f**–**k**), and ABB (**l**–**q**) of *Musa* spp. Statistical significance was calculated in comparison to transcript abundance. Error bars indicate standard error of means (SEM) from 3 biological replicates (*n* = 3). Asterisks (1, 2, and 4) indicate significant differences at *p* < 0.05, *p* < 0.01, and *p* < 0.001, respectively, using 2-way ANOVA, followed by Tukey test. (**r**) Correlation heatmap of relative expression of miRNAs between drought stress and control samples. Boxes’ color and intensity represent changes (not absolute values) in miRNA expression in leaves and roots.

qRT-PCR was conducted for all predicted 17 miRNAs, of which 6, miR530-5p, miR528-5p, miR482a, miR397a, miR160h, and miR399a, exhibited distinct expression patterns in the leaves and roots of three genomic groups (AAA, AAB, and ABB) of *Musa* spp. To characterize these miRNAs, various literature sources were reviewed, revealing that most identified *Musa* miRNAs belonged to stress-responsive groups, including miR530-5p, miR482a, miR528-5p, miR397a, and miR172i [3]. The expression levels of miRNAs in the control and stressed tissues of different groups were compared, and distinct patterns of expression profiles were observed (Figure 7). In the group of drought-sensitive banana (AAA), the relative expression of miR530-5p, miR399a, and miR528-5p in the stressed leaves was upregulated compared to the control. Whereas the levels of miR528-5p slightly increased, the expression of miR482a and miR399a in the roots was not detected. However, in AAB groups, the levels of miR528-5p, miR397-5p, and miR160h were increased in the stressed roots; interestingly, miR397a was not detected in the roots. The expression profiles of selected miRNAs in the highest drought-tolerant ABB group of *Musa* spp. were further investigated. It was observed that the levels of miR528-5p and miR482a were upregulated in the stressed roots compared to the control. Additionally, the levels of miR530-5p, miR160h, miR528-5p, miR397a, and miR399a showed similar patterns in the control leaves, where they were upregulated under control conditions. Furthermore, the generated heatmap revealed the correlated expression patterns of identified *Musa* miRNAs in the leaves and roots of *Musa* spp. (Figure 7).

**Figure 8:**
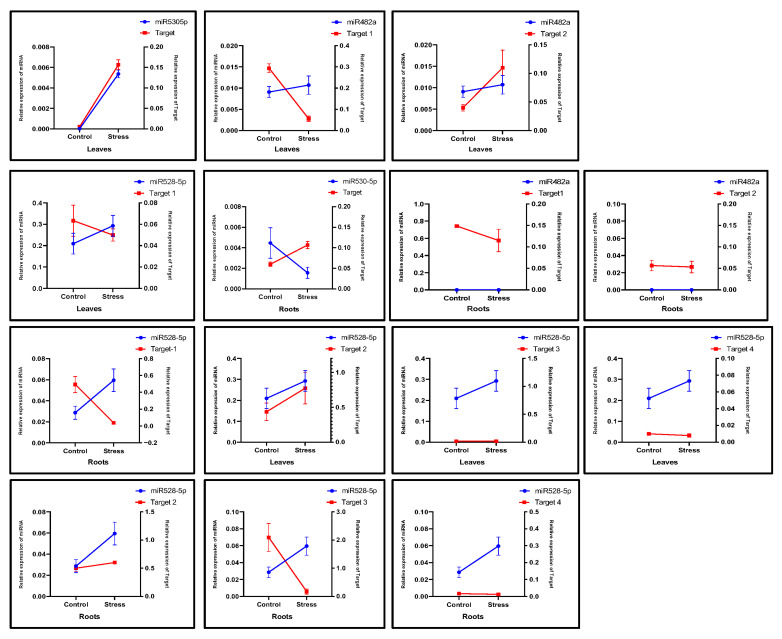
The relative expression profiles of *Musa* miRNAs, miR530-5p, miR482a, and miR528-5p, and their targets observed in the leaves and roots of the AAA group in response to drought stress. The left and right y-axes represent the relative expression (2^−ΔCt^) of miRNAs and their target genes, respectively. The blue and red lines represent the miRNAs and target genes, respectively. Three biological replicates were used. The values represent the mean, and the error bars represent the standard error of the mean.

To compare the expression of targets with that of miRNAs such as miR530-5p, miR160h, miR528-5p, miR397a, miR160h, and miR399a, qRT-PCR was performed among the genes listed in Table 3. The upregulated miRNA expression levels related to their targets should result in a corresponding decrease in target levels, indicating a reciprocal relationship. In the AAA group of *Musa* spp., the target of miR530-5p was increased in the roots, while the level of miR530-5p was downregulated (Figure 8). However, the roots showed the opposite pattern on Target-1 and Target-3 of miR528-5p. During drought stress, miR528-5p levels increased, while its target (1 and 3) levels went down in the roots. Interestingly, no such relationship was observed in the leaves, except for miR482a. The levels of target-1 of miR482a were downregulated with a small increase in the miR482a level.

**Figure 9:**
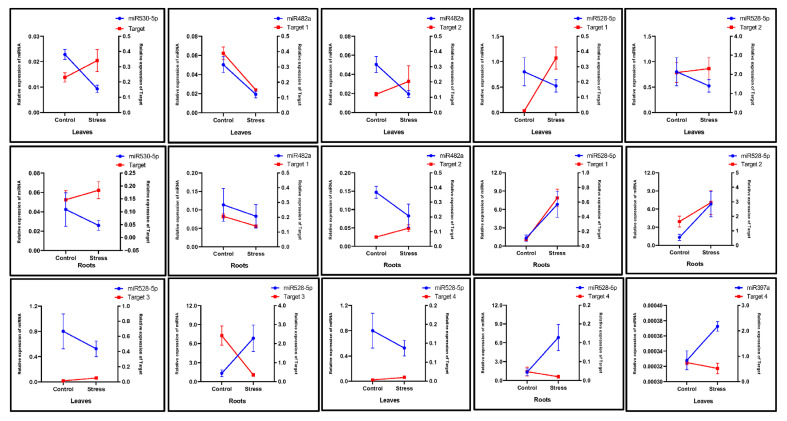
The expression profiles of *Musa* miRNAs, miR530-5p, miR482a, miR528-5p, and miR397a, and their targets observed in the leaves and roots of the AAB group in response to drought stress. The left and right y-axes represent the relative expression (2^−ΔCt^) of miRNAs and their target genes, respectively. Three biological replicates were used. The value represents the mean, and the error bars represent the standard error of the mean.

The AAB groups, which are moderately drought-tolerant, showed different expression relationships of miRNA targets in the leaves and roots. It was observed that in the leaves, the level of miRNA530-5p decreased under drought stress, while the expression of its target gene increased. Interestingly, a similar pattern was also observed in the roots (Figure 9). Likewise, the expression level of miR528-3p decreased in the leaves, and its Target-1 gene expression was increased. In contrast, the opposite pattern in the roots was observed, where miR528-5p was upregulated, and its Target-3 gene expression was downregulated. The other miRNAs failed to reveal such relationships in the leaves and roots of the AAB group of *Musa* species.

**Figure 10:**
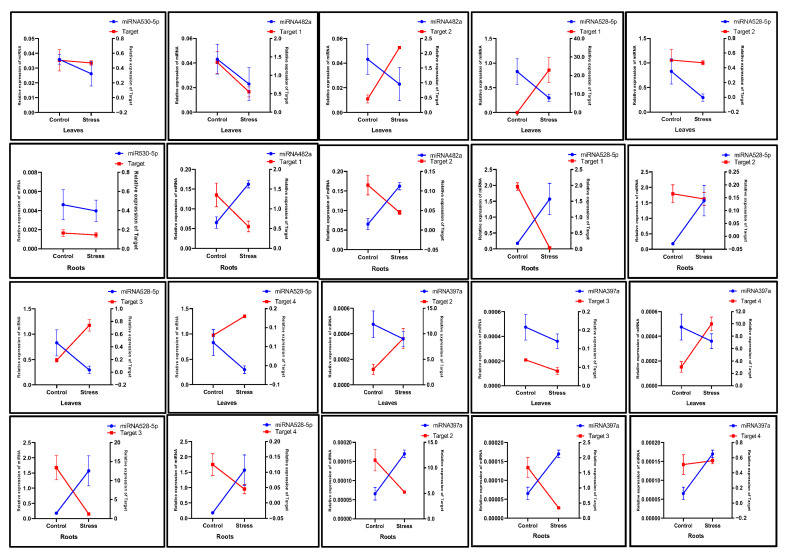
The relative expression profiles of *Musa* miRNAs, miR530-5p, miR482a, miR528-5p, and miR397a, and their target genes observed in the leaves and roots of the ABB group in response to drought stress. The left and right y-axes represent the relative expression (2^−ΔCt^) of miRNAs and their target genes, respectively. Three biological replicates were used. The value represents the mean, and the error bars represent the standard error of the mean.

The relationship between miRNAs and their target gene expression in the leaves and roots of the ABB group was also analyzed. (Figure 10). The expression levels of miR530-5p and its target gene did not reveal any changes in either tissue. Although miR482a was highly expressed in the stressed roots, its Target-1 and Target-2 genes were downregulated. Interestingly, in the stressed leaves, the expression levels of miR528-5p were decreased, while its target genes, excluding Target-2, were upregulated. In contrast, in the stressed roots, miR528-5p levels increased, while the expression levels of target genes such as Target-1, Target-3, and Target-4 were downregulated. A similar pattern was observed for miR397a in the stressed roots, where its expression was upregulated, and its Target-2 expression level was downregulated. Overall, the expression profiles of miRNAs and their corresponding target genes were distinct across different tissue types in the AAA, AAB, and ABB groups of *Musa* spp. under drought stress.

## 3. Discussion

The in silico analysis of high-throughput transcriptome resources such as ESTs and TSA contigs has become an increasingly prevalent method for miRNA identification in plant species that lack fully annotated genomes. Therefore, the in silico-based prediction of miRNAs and target gene analysis has been performed in many plant species such as *Cannabis sativa* [36], *Macrotyloma uniflorum* [14], *Oryza sativa* [37], and *Nicotiana benthamiana* [38]. Very few studies have investigated the role of miRNAs in response to different abiotic stressors, particularly in *Musa* spp. In this study, using 37,128 TSA and 45,771 EST sequences from *Musa* species, 231 non-coding sequences were identified to predict precursor miRNAs in *Musa* spp. This corroborates previous studies conducted on *Musa* spp. using ESTs and GSSs [13] as well as in *Macrotyloma uniflorum* [14] and *Ipomoea batatas* [39] with EST and TSA sequences from the gene bank. Validating the stem-loop secondary structure is a crucial step for the prediction of pre-miRNAs. Based on this, 17 precursor miRNAs with minimal folding free energy (MFE) ranging from −51.3 to −88.40 kcal mol^−1^ were identified, which is consistent with previously reported MFE values that characterize the stable miRNA structure in *Musa* spp. [11,13], as well as in *Macrotyloma uniflorum* [14], *Cannabis sativa* [36], and *Vaccinium macrocarpon* [40]. We observed 17 miRNAs belonging to 11 miRNA families such as miR169, miR156, miR482, miR528, miR397, miR399, miR160, miR530, miR172, miR166, and miR398, which were reported previously to play various roles in plant development and stress responses [3,33,35,40,41]. MicroRNAs are critical regulators of gene expression in plants, controlling either the cleavage or translation repression of their target mRNAs by binding to complementary sequences. In this study, it was identified that 11 miRNA families target 59 predicted genes, applying an e-value cutoff (≤3) to minimize false positives with the psRNATarget tool. The predicted target genes include key protein families such as Leucine-rich repeat (LRR) receptor-like serine/threonine kinases and NBS-LRR disease resistance proteins, suggesting their roles in pathogen recognition and immune signaling [41,42]. Polygalacturonase, polyphenol oxidases, and laccases are implicated in miRNA-mediated cell wall remodeling and oxidative defense [42,43,44]. Furthermore, regulatory proteins such as NAC transcription factors and RGA2 highlight miRNA involvement in developmental programming and hormone signaling [45,46,47,48]. The GO enrichment analysis revealed that the gene sets were enriched with DNA binding, oxidoreductase activity, and hydrolase activity, signifying that the miRNA targets were not only transcription factors but also a spectrum of enzymes that modulate stress responses [49,50]. The genome-wide comparison of miRNA target proteins against four model plant species provided a detailed view of conserved and lineage-specific gene families in *Musa*. Similar studies have been conducted in *Macrotyloma uniflorum*, where the target genes were conserved in different plant species [14]. It was noted that the highest cluster counts were identified in hexaploid wheat that arises from recent polyploidy events. As polyploidy events continue to occur, *Musa acuminata* may eventually shift toward a higher position among monocots in terms of gene family expansion, similar to what has been observed in hexaploid wheat. However, single-copy ortholog analysis revealed 212 gene families that arose uniquely in *Musa* lineages.

Our study revealed that, of the 17 predicted miRNAs identified, 6 (six), miR530-5p, miR528-5p, miR482a, miR397a, miR160h, and miR399a, exhibited distinct expression patterns in the leaves and roots of the AAA, AAB, and ABB *Musa* groups. Notably, many of the miRNAs are involved in several stress-specific functions, as previously reported in both monocots and dicots. For example, miR160 regulates ABA metabolism [51,52,53,54]; miR530 controls CAM genes [55] and contributes to nutrient homeostasis [56,57]; miR397a is associated with cold stress in soybean [34,58]; miR399 regulates key enzymes in ABA biosynthesis [58,59]; miR482 is associated with disease resistance [60,61,62]; and monocot-specific miR528 plays many roles, including regulating flowering time [63], responding to heavy metal stress in *Oryza sativa* [64], and participating in signal transduction in *Zea mays* [65]. In our drought stress experiment, expression analysis revealed that specific miRNAs responded to stress in a manner dependent on both *Musa* genotypes (AAA, AAB, and ABB) and tissue types (leaves vs. roots). The distinct expression profile, such as upregulation, downregulation, or even non-detection under certain conditions and groups, suggests that these specific miRNAs may play important roles in the differential drought tolerance mechanisms among the *Musa* groups. In this study, miRNA target gene analysis showed that the upregulation of miRNA expression generally corresponds to a decrease in the expression levels of their target genes. However, this relationship varies depending on the *Musa* genome groups and tissue types. Interestingly, miR528 exhibited significantly increased expression in the roots of the AAA group, while its targets, such as polyphenol oxidase and mavicyanin, were downregulated. A similar trend was observed in the AAB and ABB groups. Other miRNAs also showed distinct expression patterns across the three *Musa* genome groups. In a previous study conducted under soil moisture deficit stress, the researchers did not detect miR528; however, miR169, miR156, and miR2118 were upregulated under the same conditions. Among them, miR169 played a transcriptional regulatory role in the expression of target genes dehydrin and aquaporin [11]. Under biotic stress conditions, such as infection with *Fusarium oxysporium*, high expression levels of miR169 family members and their target genes were detected in *Musa acuminata* [66]. Another study showed that the infection of *Pseudocercospora musae* in a variety of Calcutta 4 (AA) characterized 11 conserved miRNAs and some of their target genes associated with plant defense responses [35]. Our study identified *Musa* miR397a, which was upregulated in the stressed roots of the ABB group, where its target genes Target-2 and Target-3 were downregulated. Similar findings have been reported under copper, ABA, and heat treatments in banana, and also the overexpression of native *Musa*-miR397 enhanced the overall biomass of the plants [34]. In our study, we also noted that miR528 showed the highest expression levels in both roots and leaves across different *Musa* genome groups, with different patterns of expression, further highlighting its responsiveness to stress. Interestingly, a similar transcriptomic-based gene expression study conducted on the AAA and ABB groups of *Musa* spp. under drought stress revealed differential expression patterns across the two genomic groups [67]. Further investigations are required to validate the cleavage sites of target transcripts using 5′ RLM-RACE and sequencing. Although some studies have explored miRNA expression under stress conditions in *Musa*, few have combined genome-wide in silico analysis with experimental validation across multiple *Musa* genome groups. In this study, we examined drought-responsive miRNAs and their target genes in the AAA, AAB, and ABB groups, identifying several unique miRNAs with potential regulatory roles by integrating in silico predictions with expression profiling.

## 4. Materials and Methods

### 4.1. Datasets for Query and miRNA Resources

A total of 38,182 shotgun-assembled contigs of transcriptome sequences (TSAs) from the set GABH01000001_GABH01038183 were downloaded from the European Nucleotide Archive (ENA: https://www.ebi.ac.uk/ena/browser/view/GABH01000000, accessed on 16 October 2024). To represent the query sequences for miRNA homologs, 45,771 NCBI ESTs (expressed sequence tags), along with a library of drought stress EST sequences of *Musa* spp. and TSA sequences, were used. A total of 9988 previously known mature viridiplantae miRNAs (Released 22.1, 13.11.2018) were retrieved from miRBase [68] (https://www.mirbase.org/, accessed on 9 October 2024) and clustered using CD-HIT EST version 4.8.1 [69] with a sequence identity threshold value of 1.0 (100). Following clustering, 5510 non-redundant miRNA sequences were used as reference sequences by constructing a local nucleotide sequence database to identify the homologs of *Musa* miRNAs.

### 4.2. Bioinformatic Tools Used for Analysis

To search the homology-based conserved predicted miRNAs of *Musa* spp., the query sequences of TSAs and ESTs were processed against the local database, which was carried out using the NCBI blastn [70] (version 2.14.1) alignment tool with an expectation value cutoff of 0.001. CD-HIT EST was used to generate non-redundant miRNA sequences for the analysis. The secondary structure and free energy analyses were conducted using the Mfold web server V3.6 [71] (https://www.unafold.org/mfold/applications/rna-folding-form.php, accessed on 11 December 2024) to predict the precursor miRNA (pre-miRNA) of *Musa* spp.; miRNA targets were assembled and analyzed using the psRNA target server (version 2.0) [72]. The orthologous clusters from target genes were identified and annotated using the OrthoVenn3 platform [73]. Additionally, OmicsBox (version 3.4.6) software was utilized to determine the functional roles and classify gene ontology (GO) terms related to molecular functions, biological functions, and cellular components [74].

### 4.3. Identification of Predicted miRNA Homologs

The predicted miRNA homologs were identified and analyzed using the TSA and EST sequences derived from the ENA and NCBI databases. The non-redundant clustered miRNAs generated through CD-HIT and their representative sequences were employed to create a local database using Blast version 2.14.1. The query sequences of TSAs and ESTs were subjected to nucleotide blast (blastn) against the reference sequences of plant miRNAs with the following setup parameters [75]: (i) the length of mature miRNA sequence ≥ 18 nt without gap, (ii) no more than 2 nt mismatches allowed, and (iii) an E-value cutoff of 0.001. The generated filtered sequences were further utilized to remove protein-coding sequences using NCBI blastx against the non-redundant (nr) database. Only non-coding sequences were exploited for secondary structure prediction and validation.

### 4.4. Prediction of Secondary miRNA Structure and Validation

All non-coding sequences with maximum stringency were subjected to the prediction of the secondary structure using the algorithm in the Mfold server version 2.3. The pre-miRNAs were extracted using the following method [76] of a sliding window of 100 nt size (moving in increments of approximately 20 nt) from ~80 nt upstream of the beginning of mature miRNA to ~80 nt downstream of the miRNA. The following parameters were used in Mfold analysis: (i) folding temperature 37 °C, (ii) ionic conditions of 1 M NaCl without divalent ions, (iii) linear RNA, (iv) percent suboptimal number 5, and (v) maximum interior/bulge loop size 30. The following criteria [71] were used for detecting the precursor miRNA structure: (i) There can be no more than 3 nt mismatches between predicted mature miRNA and known plant miRNA. (ii) Precursor miRNA sequences can fold into an accurate hairpin stem-loop secondary structure, and the mature identified miRNAs are located in one arm of the corresponding hairpin stem-loop. (iii) The value of the AU% of pre-miRNA ranges from 30% to 70%. (iv) There are less than six mismatches between mature miRNA and its opposite star miRNA sequence. (v) The secondary structure of pre-miRNA should have a high negative MFE (≤−20 kcal mol^−1^) and MFEI value.

### 4.5. Phylogenetic Analysis

The conserved nature and phylogenetic relationship of putative precursor miRNAs were employed for phylogenetic analysis along with the available precursor miRNA sequences from different plant species retrieved from miRBase. The sequences were aligned with ClustalW version 2.1, and the output was further used in MEGA version 12.0 [77] and iTOL to generate a tree.

### 4.6. Prediction and Functional Annotation of miRNA Targets

The newly identified miRNAs, along with *Musa* reference sequences, were used to identify their predicted target genes. The online psRNA Target version 2.0 was used with the following parameters: (i) the scoring maximum expectation was 3.0, (ii) the length of HSP scoring was 20, (iii) translational inhibition ranged from 9 to 11 nt, (iv) the length of the flanking region around the target site for accessibility analysis was kept 17 bp upstream and 13 bp downstream, and (v) the target accessibility of the mRNA target sites allowed the maximum energy to unpair the target site (UPE) was kept at 25.0. The identified target mRNAs were analyzed for gene ontology (GO) term analysis using blast2go on the OmicsBox platform to determine their functional annotations, molecular functions, biological processes, and cellular components.

### 4.7. Analyses of Orthologous Target Genes in Different Plant *spp*.

The annotation of miRNA target proteins to analyze their converseness, OrthoVenn3, 2022 [73] (https://orthovenn3.bioinfotoolkits.net/home, accessed on 13 December 2024), was used for comparisons with other plant species. The protein sequence data of miRNA targets was uploaded onto the web server with default parameters in the OrtholoMCL algorithm with an E-value of 1e-2 and an inflation value of 1.50. In addition, CAFE5 [78] was employed to estimate expansion or contraction depending on the size and evolutionary period of the species’ gene family in the same platform.

### 4.8. Plant Materials and Drought Stress

Three different groups of *Musa* spp., Chinese Cavendish (AAA, Acc. No. ITC0547), Silk (AAB, Acc. No. ITC0348), and Monthan (ABB, Acc. No. ITC1724), were sourced from the International Transit Centre, Laboratory of Tropical Crop Improvement, Leuven, Belgium (Biodiversity International, Belgium) https://www.biw.kuleuven.be/biosyst/cropbiotechnics/tropical, accessed on 23 February 2024. The plants were grown in a CONVIRON TC80 (Conviron, Pembina, ND, USA) growth chamber under controlled conditions at the Department of Natural Sciences, Bowie State University, for use in this study. Stress experiments were conducted in the Tropical Greenhouse at Bowie State University under controlled temperatures ranging from 24 °C to 26 °C. Previous studies have induced drought stress in *Musa* spp. using 17-day mannitol treatment [79], withholding water for 12–24 [11,79] days and even conducting dehydration on filter paper for 0–12 h [80,81]. Based on these precedents, two-and-a-half-month-old plants were subjected to drought stress (experimental) by withholding water for 20 days. Control plants received water regularly, and the soil moisture content was measured with a moisture meter (Model HG01-TX-Temp-V05, Dr.meter, Newark, CA, USA). Further, an electrolyte leakage assay [82] was conducted by excising leaves from the control and experimental samples. Conductivity was measured by a conductivity tester (Model-EC20, Apera Instruments, Columbus, OH, USA).

### 4.9. RNA Isolation and Quantitative Expression Analyses of miRNAs and Their Targets

Leaves and root tissues from selected banana and plantain plants were collected for total RNA extraction. Samples from both control and drought-stressed plants across three *Musa* groups were harvested and stored at −80 °C until RNA isolation. Total RNA was isolated using the buffer described in our recent protocol and fused with a *mirVana* kit (AM1560, Invitrogen, Vilnius, Lithuania) to enrich the small RNAs. To study miRNA expression, 2 µg of total RNA was reverse-transcribed using Superscript IV reverse transcriptase (SSIV-RT, Invitrogen) and stem-loop primers [83]. A reverse transcription quantitative PCR (RT-qPCR) was performed as per the protocol developed by the Varkonyi-Gasic group [84] with miRNA-specific universal forward primers and reverse primers. Additionally, to study miRNA target gene expression, complementary DNA (cDNA) was synthesized using Oligo (dT) primers with SSIV-RT. Table S3 provides a list of all mentioned primers utilized for this study. PCRs were prepared with TB Green Premix Ex Taq II (Takara-RR82WR) and incubated at 95 °C for 30 s, followed by 40 cycles of 95 °C for 10 s, gene-specific annealing temperature for 15 s, and extension at 72 °C for 20 s was performed in CFX96 Touch Real-Time PCR Detection System (BioRad-C1000, Hercules, CA, USA). All reactions were performed with technical triplicates, and normalization was carried out using endogenous reference genes *MaUBQ2* (for mRNA) and *MaU6* (for miRNA). Melt curve analysis was performed to verify PCR specificity, and data analysis was conducted using the 2^−ΔCt^ method [85].

### 4.10. Statistical Analysis

Graphs were generated using GraphPad Prism version 8.0.1, and data were analyzed by a two-way ANOVA followed by Tukey’s HSD post hoc test to determine individual statistical differences. Error bars represent the standard error of means (SEM), and significance levels are indicated by one, two, or three asterisks for *p* < 0.05, <0.01, and <0.001, respectively.

## 5. Conclusions

In this study, we identified 17 putative conserved miRNAs and 59 target genes through in silico analysis using transcriptome shotgun assemblies (TSAs) and expressed sequence tags (ESTs) from the *Musa* species. Notably, six conserved *Musa* miRNAs exhibited distinct spatiotemporal expression patterns in three *Musa* groups under drought stress. Target gene analysis revealed predominant involvement in the metabolism, stress response, and signal transduction pathways in plants. An inverse expression relationship was observed in miRNA-mRNA modules, wherein specific miRNAs were upregulated while their corresponding target genes were downregulated in the leaves and roots under drought conditions. Of particular interest, miR528 and its targets demonstrated strong stress responsiveness in roots, suggesting their potential as a key regulatory candidate for studying the role of drought tolerance mechanisms, particularly in *Musa* spp. Future functional studies are needed to elucidate the regulatory interaction between miRNAs and their targets, with the goal of improving drought resilience in *Musa* spp.

## Figures and Tables

**Table 1:** Characterization of predicted potential miRNAs identified from TSA and EST sequences. Numerical values include size of predicted precursor lengths, percentage of AU/GC content, and measurement of secondary structure thermodynamic stability as minimal free folding energy (MEF) and adjusted minimal folding free energy of precursor miRNAs from *Musa* spp.

EST/TSA ID.	miRNA Families	LE/T	LP	AU%	GC%	A	C	G	U/T	MFE	AMFE	MFEI
FL647992	miR169j-5p	571	110	44.91	55.09	40	58	34	35	−55.70	−50.630	0.92
JK538379	miR156f	229	127	49.28	50.72	30	41	30	39	−58.40	−45.980	0.91
ES434836	miR156a-3p	789	91	41.21	58.79	27	49	51	55	−76.80	−84.400	1.44
DN238517	miR482a	236	65	38.12	61.88	29	55	51	46	−88.40	−136.000	2.20
FL667486	miR528-5p	674	62	45.56	54.44	52	38	67	23	−66.00	−106.000	1.95
FL666615	miR397a	586	96	56.39	43.61	38	28	37	30	−58.50	−60.930	1.40
FL666459	miR399a	561	136	44.12	55.88	35	50	38	47	−55.80	−41.029	0.73
FL666054	miR160h	392	96	37.57	62.43	44	49	53	35	−71.30	−74.270	1.19
FL659295	miR530-5p	807	65	68.33	31.67	29	29	33	29	−46.40	−71.380	2.25
FL666615	miR397-5p	586	78	60.77	39.23	35	28	37	30	−56.60	−72.560	1.85
FL647629	miR169a	721	116	32.22	67.78	54	39	42	45	−67.00	−57.760	0.85
GABH01012340	miR166	4052	153	47.25	52.75	29	56	40	57	−59.8	−39.085	0.71
GABH01015288	miR156j	4850	81	44.79	55.21	42	34	72	44	−87.7	−108.272	2.13
GABH01000646	miR398a-3p	1536	113	50.83	49.17	50	40	49	42	−63.5	−56.195	0.96
GABH01012340	miR166e-3p	4052	141	47.51	52.49	29	55	40	57	−59.9	−42.482	0.69
GABH01012340	miR166h-3p	4052	142	48.07	51.93	30	55	39	57	−60.4	−42.535	0.78
GABH01002598	miR172i	2269	160	51.11	48.89	39	35	53	53	−51.3	−32.063	0.74

LE/T: length of ESTs and TSA; LP: length of precursor miRNA; MFE: minimal folding free energy (kcal mol^−1^); AMFE: adjusted minimal folding free energy.

**Table 2:** Predicted *Musa* miRNA sequences with length (LM) and guide strand position with their homologs from several known plant species.

*Musa* miRNA	miRNA Homolog	Mature miRNA Sequences	LM	Loc	Strand
miR169j-5p	ata-miR169j-5p	UAGCCAAGGAUGAUUUGCCUGUG	23	5′	−
miR156f	gma-miR156f	UUGACAGAAGAGAGAGAGCACA	22	5′	−
miR156a-3p	vca-miR156a-3p	UGCUCACUUCUCUUUCUGUCAG	21	3′	+
miR482a	fve-miR482a	UCUUUCCAAUUCCUCCCAUGCC	22	3′	+
miR528-5p	osa-miR528-5p	UGGAAGGGGCAUGCAGAGGAG	21	5′	+
miR397a	bna-miR397a	UCAUUGAGUGCAGCGUUGAUGU	21	5′	+
miR399a	osa-miR399a	UGCCAAAGGAGAAUUGCCCUG	21	3′	+
miR160h	ptc-miR160h	UGCCUGGCUCCCUGCAUGCCA	21	5′	−
miR530-5p	osa-miR530-5p	UGCAUUUGCACCUGCACCUA	20	5′	+
miR397-5p	stu-miR397-5p	AUUGAGUGCAGCGUUGAUGAC	20	5′	+
miR169a	pab-miR169a	UCAGCCAAGAAUGACUUGCCC	20	5′	−
miR160	ctr-miR166	UCGGACCAGGCUUCAUUCCCCC	22	5′	+
miR156j	cme-miR156j	GUUGACAGAAGAGAGUGAGCAC	22	5′	+
miR398a-3p	ath-miR398a-3p	UGUGUUCUCAGGUCACCCCUU	21	3′	−
miR166e-3p	bdi-miR166e-3p	CUCGGACCAGGCUUCAUUCCC	21	3′	+
miR166h-3p	gma-miR166h-3p	UCUCGGACCAGGCUUCAUUCC	21	3′	+
miR172i	ptc-miR172i	AGAAUCCUGAUGAUGCUGCAA	20	3′	+

**Table 3:** Description of putative target genes of selected *Musa* microRNAs depicting confidence of target interaction (E-value), type of inhibition, and biological functions.

miRNA Family	Name of Targets	Target Description	E-Value	Inhibition	Functions
miR482a	Target-1	NBS-LRR class resistance protein (Fragment)	2	Cleavage	Defense Response
	Target-2	Disease resistance protein RGA2, putative, expressed	3	Translation	Defense Response
miR528-5p	Target-1	Polyphenol oxidase, chloroplastic	1	Cleavage	Stress Responses
	Target-2	Putative Leucyl-tRNA synthetase, cytoplasmic	2.5	Cleavage	Metabolism
	Target-3	Mavicyanin	3	Cleavage	Transport
	Target-4	Putative serine/threonine protein kinase fray2	3	Cleavage	Signal Transduction
miR397a	Target-1	Laccase-4	1.5	Cleavage	Stress Responses
	Target-2	Putative S-(hydroxymethyl)glutathione dehydrogenase	2	Cleavage	Stress Responses
	Target-3	Serine carboxypeptidase-like 35	3	Cleavage	Stress Responses
	Target-4	Putative NAC domain-containing protein 74	3	Cleavage	Transcription Factor
miR530-5p	Target	Tetratricopeptide repeat domain-containing protein, expressed	2.5	Cleavage	Cellular Process

## Data Availability

All data generated and analyzed during this study are presented in this article.

## Supplementary Materials

The supporting information can be downloaded at https://www.mdpi.com/article/10.3390/ijms26136385/s1.
