# Supplementary material for: In Silico Genome-Wide Profiling of Conserved miRNAs in AAA, AAB, and ABB Groups of Musa spp.: Unveiling MicroRNA-Mediated Drought Response
Source: Int J Mol Sci. 2025 Jul 2;26(13):6385. doi: 10.3390/ijms26136385 (PMC12250125; doi:10.3390/ijms26136385)
Supplement: Supplementary file 1 [file ijms-26-06385-s001.zip › Figure S1_moisture content.pdf]

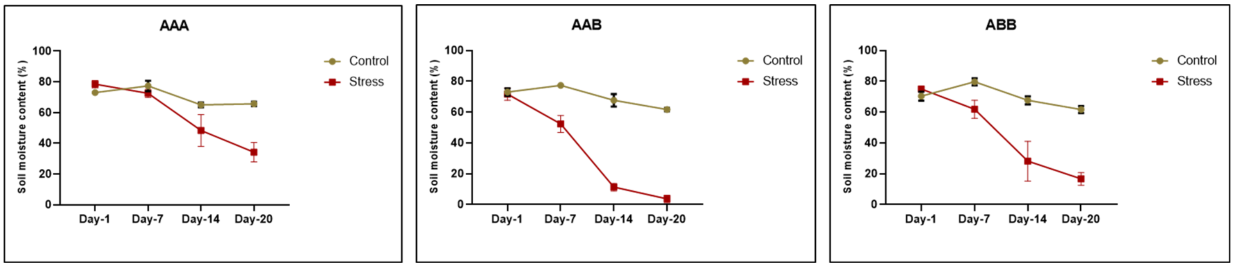

Figure S1: The percentage of soil moisture content under control and drought stress conditions across AAA, AAB, and ABB genomic groups of *Musa* spp. The decreased moisture content indicates the establishment and progression of drought stress during the experimental period.
