# Supplementary material for: In Silico Genome-Wide Profiling of Conserved miRNAs in AAA, AAB, and ABB Groups of Musa spp.: Unveiling MicroRNA-Mediated Drought Response
Source: Int J Mol Sci. 2025 Jul 2;26(13):6385. doi: 10.3390/ijms26136385 (PMC12250125; doi:10.3390/ijms26136385)
Supplement: Supplementary file 1 [file ijms-26-06385-s001.zip › Table S2_ List of target genes.pdf]

**Table S2**. Description of putative target genes of candidate microRNAs identified using ps RNAtarget based on Banana genome database.

| miRNA family | Sequences | Target Accessions | Target Description | E Value | Inhibition | Functions |
|---|---|---|---|---|---|---|
| miR169j-5p | UAGCCAAGGAGACUGCCCA | GSMUA_Achr3T31490_001 | Probable LRR receptor-like serine/threonine-protein kinase MRH1 | 2.5 | Cleavage | Stress Responses |
| miR156f | GCUCUCUAUGCUUCUGUCAUCA | GSMUA_Achr3T19540_001 | SNF1-related protein kinase regulatory subunit gamma 1 | 3 | Cleavage | Metabolism |
| | | GSMUA_Achr11T11210_001 | Polygalacturonase | 3 | Cleavage | Metabolism |
| miR156a-3p | UGCUCACUUCUCUUCCUGUCAG | GSMUA_Achr9T00190_001 | Putative Receptor protein kinase CLAVATA1 | 2 | Cleavage | Signal Transduction |
| | | GSMUA_Achr10T30820_001 | ELMO/CED-12 family protein, putative, expressed | 2.5 | Cleavage | Metabolism |
| | | GSMUA_Achr9T29620_001 | Putative Abscisic acid 8'-hydroxylase 3 | 3 | Cleavage | Stress Responses |
| | | GSMUA_Achr2T05450_001 | Putative Transcription factor BIM2 | 3 | Translation | Transcription Factor |
| | | GSMUA_Achr4T17780_001 | Putative Indole-3-acetate beta-glucosyltransferase | 3 | Cleavage | Growth Development |
| | | GSMUA_Achr4T26970_001 | E3 ubiquitin-protein ligase BRE1-like 1 | 3 | Cleavage | Transcriptional regulation |
| | | GSMUA_Achr5T10800_001 | ABC transporter G family member 11 | 3 | Cleavage | Stress Responses |
| | | GSMUA_Achr11T11020_001 | Putative Pentatricopeptide repeat-containing protein At5g66500, mitochondrial | 3 | Cleavage | Growth development |
| | | GSMUA_Achr8T22880_001 | Putative PHD finger protein At1g33420 | 3 | Cleavage | Metabolism |
| miR482a | CUUUCCAAUACCUCCCAUGCC | GSMUA_Achr6T32050_001 | NBS-LRR class resistance protein (Fragment) | 2 | Cleavage | Defense Response |
| | | GSMUA_Achr7T21400_001 | disease resistance protein RGA2, putative, expressed | 3 | Translation | Defense Response |
| miR528-5p | UGGAAGGGGCAUGCAGAGGAG | GSMUA_Achr7T03450_001 | Polyphenol oxidase, chloroplastic | 1 | Cleavage | Stress Responses |
| | | GSMUA_AchrUn_randomT25220_001 | Putative Polyphenol oxidase A1, chloroplastic | 2.5 | Cleavage | Stress Responses |
| | | GSMUA_Achr8T21510_001 | Phospholipid-transporting ATPase 1 | 2.5 | Cleavage | Transport |
| | | GSMUA_Achr7T23600_001 | Putative Leucyl-tRNA synthetase, cytoplasmic | 2.5 | Cleavage | Metabolism |
| | | GSMUA_Achr8T33600_001 | Probable indole-3-acetic acid-amido synthetase GH3.6 | 2.5 | Translation | Growth Development |
| | | GSMUA_Achr6T29640_001 | Mavicyanin | 3 | Cleavage | Transport |
| | | GSMUA_Achr3T04830_001 | Putative Serine/threonine-protein kinase fray2 | 3 | Cleavage | Signal Transduction |
| | | GSMUA_Achr6T29370_001 | Polyphenol oxidase, chloroplastic | 3 | Cleavage | Stress Responses |
| | | GSMUA_Achr6T08010_001 | Serine palmitoyltransferase 2 | 3 | Cleavage | Metabolism |

| | | | | | | |
|---|---|---|---|---|---|---|
| miR397a | CAUCAUUG AGUGCAGC GUUGA | GSMUA_Achr 2T16380_001 | Laccase-4 | 1.5 | Cleavage | Stress Responses |
| | | GSMUA_Achr 5T25890_001 | Putative S-(hydroxymethyl)glutathione dehydrogenase | 2 | Cleavage | Stress Responses |
| | | GSMUA_Achr 11T24220_001 | Laccase-22 | 2.5 | Cleavage | Stress Responses |
| | | GSMUA_Achr 6T22750_001 | Endoglucanase 8 | 2.5 | Cleavage | Metabolism |
| | | GSMUA_Achr 1T11960_001 | Mitogen-activated protein kinase 10 | 2.5 | Cleavage | Metabolism |
| | | GSMUA_Achr 6T33870_001 | Laccase-25 | 3 | Translati on | Stress Responses |
| | | GSMUA_Achr 3T15320_001 | Laccase-2 | 3 | Translati on | Stress Responses |
| | | GSMUA_Achr 6T34080_001 | Laccase-17 | 3 | Cleavage | Stress Responses |
| | | GSMUA_Achr 4T29430_001 | Serine carboxypeptidase-like 35 | 3 | Cleavage | Stress Responses |
| | | GSMUA_Achr 11T08770_001 | Putative ZOS1-20 - $C_2H_2$ zinc finger protein, expressed | 3 | Cleavage | Stress Responses |
| | | GSMUA_Achr 5T23620_001 | Putative NAC domain-containing protein 74 | 3 | Cleavage | Transcription factor |
| miR399a | UGCCAAAG GAGAAUUG CCCUG | GSMUA_Achr 8T15350_001 | DEAD-box ATP-dependent RNA helicase 37 | 3 | Cleavage | Transcription al regulation |
| | | GSMUA_Achr 10T11810_001 | Putative [Protein-PII] uridylyltransferase | 3 | Cleavage | Signal Transduction |
| miR160h | UGCCUGGC UCCCUGCA UGCCA | GSMUA_Achr 6T18900_001 | Putative Auxin response factor 22 | 0 | Cleavage | Growth Development |
| | | GSMUA_Achr 9T29480_001 | Putative Auxin response factor 17 | 0 | Cleavage | Growth Development |
| | | GSMUA_Achr 5T18540_001 | Auxin response factor 18 | 1 | Cleavage | Growth Development |
| miR530-5p | CUGCAUUU GCACCUGC ACCU | GSMUA_Achr 8T09010_001 | tetratricopeptide repeat domain containing protein, expressed | 2.5 | Cleavage | Cellular Process |
| miR169a | GGCAAGUC AUUCUUGG CUGA | GSMUA_Achr 3T18240_001 | WD repeat-containing protein 44, putative, expressed | 3 | Cleavage | Growth Development |
| miR166 | UCGGACCA GGCUUCAU UCCCCC | GSMUA_Achr 8T02960_001 | Homeobox-leucine zipper protein HOX32 | 1.5 | Cleavage | Transcription Factor |
| | | GSMUA_Achr 5T18530_001 | Homeobox-leucine zipper protein HOX9 | 2 | Cleavage | Transcription Factor |
| | | GSMUA_Achr 8T20090_001 | Homeobox-leucine zipper protein ATHB-15 | 2 | Cleavage | Transcription Factor |
| | | GSMUA_Achr 7T01060_001 | Homeobox-leucine zipper protein HOX32 | 2 | Cleavage | Transcription Factor |
| miR156j | GUUGACAG AAGAGAGU GAGCAC | GSMUA_Achr 5T22630_001 | Squamosa promoter-binding-like protein 12 | 1 | Cleavage | Transcription Factor |
| | | GSMUA_Achr 9T29330_001 | Putative OsSPL16 - SBP-box gene family member, expressed | 2.5 | Cleavage | Transcription Factor |
| | | GSMUA_Achr Un_randomT1 0030_001 | Histidine kinase 3 | 3 | Cleavage | Signal Transduction |

| | | | | | | |
|---|---|---|---|---|---|---|
| miR398a-3p | UGUGUUCUCAGGUCACCCCUU | GSMUA_AchrUn_randomT03810_001 | Putative UDP-galactose transporter homolog 1 | 3 | Cleavage | Transport |
| miR166e-3p | CUCGGACCAGGCUUCAUUCCC | GSMUA_Achr8T02960_001 | Homeobox-leucine zipper protein HOX32 | 1.5 | Cleavage | Transcription Factor |
| | | GSMUA_Achr5T18530_001 | Homeobox-leucine zipper protein HOX9 | 2 | Cleavage | Transcription Factor |
| | | GSMUA_Achr8T20090_001 | Homeobox-leucine zipper protein ATHB-15 | 2 | Cleavage | Transcription Factor |
| | | GSMUA_Achr8T02070_001 | Putative 2-oxoglutarate dehydrogenase, mitochondrial | 3 | Cleavage | Metabolism |
| miR166h-3p | UCUCGGACCAGGCUUCAUUCC | GSMUA_Achr8T02960_001 | Homeobox-leucine zipper protein HOX32 | 3 | Cleavage | Transcription Factor |
| miR172i | AGAAUCCUGAUGAUGCUGCAA | GSMUA_Achr1T09830_001 | PHAP2A protein | 0 | Cleavage | Stress Responses |
| | | GSMUA_Achr8T18550_001 | Floral homeotic protein APETALA 2 | 1 | Cleavage | Development |
| | | GSMUA_Achr11T23870_001 | AP2 domain-containing protein, expressed | 1 | Cleavage | Transcription Factor |
| | | GSMUA_Achr6T33140_001 | Putative Ethylene-responsive transcription factor RAP2-7 | 1 | Cleavage | Transcription Factor |
| | | GSMUA_Achr5T18530_001 | Homeobox-leucine zipper protein HOX9 | 2.5 | Cleavage | Transcription Factor |
