# Supplementary material for: In Silico Genome-Wide Profiling of Conserved miRNAs in AAA, AAB, and ABB Groups of Musa spp.: Unveiling MicroRNA-Mediated Drought Response
Source: Int J Mol Sci. 2025 Jul 2;26(13):6385. doi: 10.3390/ijms26136385 (PMC12250125; doi:10.3390/ijms26136385)
Supplement: Supplementary file 1 [file ijms-26-06385-s001.zip › Table S3_List of primers.pdf]

Table S3: List of the stem-loop RT primers, and target genes primer sequences used for qRT-PCR.

Name	Primer sequences (5′-3′)
miR169jSLP	GTCGTATCCAGTGCAGGGTCCGAGGTATTCGCACTGGATACGACTGGGCA
miR169FP	GACACGTAGCCAAGGAGA
miR156fSLP	GTCGTATCCAGTGCAGGGTCCGAGGTATTCGCACTGGATACGACTGATGA
miR156fFP	GCAACGGCTCTCTATGCT
miR156a-3p	GTCGTATCCAGTGCAGGGTCCGAGGTATTCGCACTGGATACGACCTGACA
miR156a-3pFP	GCAACGTGCTCACTTCTC
miR482aSLP	GTCGTATCCAGTGCAGGGTCCGAGGTATTCGCACTGGATACGACGGCATG
miR482aFP	CGGCGGCTTTCCAATACC
miR528-5pSLP	GTCGTATCCAGTGCAGGGTCCGAGGTATTCGCACTGGATACGACCTCCTC
miR528-5pFP	GACCAGTGGAAGGGGCAT
miR397aSLP	GTCGTATCCAGTGCAGGGTCCGAGGTATTCGCACTGGATACGACTCAACG
miR397aFP	CGGCGGCATCATTGAGTG
miR399aSLP	GTCGTATCCAGTGCAGGGTCCGAGGTATTCGCACTGGATACGACCAGGGC
miR399aFP	GCAACGTGCCAAAGGAGA
miR160hSLP	GTCGTATCCAGTGCAGGGTCCGAGGTATTCGCACTGGATACGACTGGCAT
miR160hFP	ATCGTATGCCTGGCTCCC
miR530-5pSLP	GTCGTATCCAGTGCAGGGTCCGAGGTATTCGCACTGGATACGACAGGTGC
miR530FP	TGTACCCTGCATTTGCAC
miR397-5pSLP	GTCGTATCCAGTGCAGGGTCCGAGGTATTCGCACTGGATACGACTCAACG
miR397-5p FP	AGCTCGCATCATTGAGTG
miR169aSLP	GTCGTATCCAGTGCAGGGTCCGAGGTATTCGCACTGGATACGACTCAGCC
miR169aFP	GCAACGGGCAAGTCATTC
U6SLP	GTCGTATCCAGTGCAGGGTCCGAGGTATTCGCACTGGATACGACTTGGAC
U6FP	GACACGCACAAATCGAGAAATG
miRNA398-3pSLP	GTCGTATCCAGTGCAGGGTCCGAGGTATTCGCACTGGATACGAAAGGGG
miRNA398-3pFP	CGGCGGTGTGTTCTCAGG
miR166e-3pSLP	GTCGTATCCAGTGCAGGGTCCGAGGTATTCGCACTGGATACGAGGGAAT
miR166e-3pFP	TGTACCCTCGGACCAGGC
miR166h-3pSLP	GTCGTATCCAGTGCAGGGTCCGAGGTATTCGCACTGGATACGAGGAATG
miR166h-3pFP	GCA ACG TCT CGG ACC AGG
miR172iSLP	GTCGTATCCAGTGCAGGGTCCGAGGTATTCGCACTGGATACGATTGCAG
miR172iFP	CGGCGGAGAATCCTGATG
Universal RP	CCAGTGCAGGGTCCGAGGTA
nbrFP_Target-1	CGTGGCAGGTATTGGGAAGACT
nbrRP_Target-1	CCTTGTCTTGGCAAGCCTTTCC
rga2FP_Target-2	TGTTCTGTCAGCCTTCATGC
rga2RP_Target-2	TGCCTCACTGACTTGTCCTG
poloxFP_Target-1	CCGATGACCCTCGCAACTTCA
poloxRP_Target-1	CGTTGAAGTAGAGGTACAAGCGG
leucFP_Target-2	GGTGATGAGTGTGTTGTGGCT
leucRP_Target-2	GTCCAAAAGACCGTGAACATGCC
mavFP_Target-3	ATCATGGGGAGCCCGAACTA
mavRP_Target-3	TGGTCTTGATCGTGATGGTGTC
srtrFP_Target-4	GGCACACTGCTCCTTTGTGAA
srtrRP_Target-4	GTGAATGTGCCCTTGGTGATGAA
lac4FP_Target-1	GCACTTGAGAATGGTGGAGAG C
lac4RP_Target-1	AACGGTGACGGTGTGGTTGG
gluFP_Target-2	TCAGGAGGACCTTTGTGAGGC
gluRP_Target-2	GCCATTCCACCCATGCTGTAC
capepFP_Target-3	GAGCAAACTTGTCGCAGCCC

capepRP_Target-3	AGTGACATTGGCGTGCAGTG
nacFP_Target-4	GGACCTACCTGCAAAATGTCGT
nacRP_Target-4	AACTTGACATTCCGATCTCTCCC
tettriFP_Target	CCAGCCACAGAAGGCAGTATT
tettriRP_Target	TAGCAAAAGGCACTGTGCATG
p2FP_Target	TCGTGACCCGCCAATTCTTTC
p2RP_Target	ATTCCACCGACCACCACCAC
Macu_Ubq2-Fw	AGAGAGATGCTGCAAAATCCA
Macu_Ubq2-Rv	CCAGCTGTCTGCTCTTGTTCT
